# Effect of the Geometrical Constraints to the Wenner Four-Point Electrical Resistivity Test of Reinforced Concrete Slabs

**DOI:** 10.3390/s21134622

**Published:** 2021-07-05

**Authors:** Kevin Paolo V. Robles, Jurng-Jae Yee, Seong-Hoon Kee

**Affiliations:** 1 Department of ICT Integrated Ocean Smart Cities Engineering, Dong-A University, Busan 49304, Korea; kpvrobles@donga.ac.kr (K.P.V.R.); jjyee@dau.ac.kr (J.-J.Y.); 2 National Core Research Center for Disaster-Free and Safe Ocean Cities Construction, Dong-A University, Busan 49304, Korea

**Keywords:** electrical resistivity of concrete, geometrical constraint, reinforcing steel, saturation

## Abstract

The main objectives of this study are to evaluate the effect of geometrical constraints of plain concrete and reinforced concrete slabs on the Wenner four-point concrete electrical resistivity (ER) test through numerical and experimental investigation and to propose measurement recommendations for laboratory and field specimens. First, a series of numerical simulations was performed using a 3D finite element model to investigate the effects of geometrical constraints (the dimension of concrete slabs, the electrode spacing and configuration, and the distance of the electrode to the edges of concrete slabs) on ER measurements of concrete. Next, a reinforced concrete slab specimen (1500 mm (width) by 1500 mm (length) by 300 mm (thickness)) was used for experimental investigation and validation of the numerical simulation results. Based on the analytical and experimental results, it is concluded that measured ER values of regularly shaped concrete elements are strongly dependent on the distance-to-spacing ratio of ER probes (i.e., distance of the electrode in ER probes to the edges and/or the bottom of the concrete slabs normalized by the electrode spacing). For the plain concrete, it is inferred that the thickness of the concrete member should be at least three times the electrode spacing. In addition, the distance should be more than twice the electrode spacing to make the edge effect almost negligible. It is observed that the findings from the plain concrete are also valid for the reinforced concrete. However, for the reinforced concrete, the ER values are also affected by the presence of reinforcing steel and saturation of concrete, which could cause disruptions in ER measurements.

## 1. Introduction

Civil infrastructure systems, such as buildings, bridges, and pavements, are the backbone of modern society and the global economy growth, leading to the sustainable development of several countries [1]. Concrete is among the most widely used materials for the construction of the Civil infrastructure systems because of its predominant characteristics such as high durability, excellent plasticity, waterproofness, and cheap cost relative to other construction materials [2,3]. The use of concrete represents about 65% of all the building materials in the world [4]. This leads to overproduction of cement that results to environmental issues such as water pollution [5], carbon dioxide emission [6], and large consumption of raw materials and energy. It is necessary to improve the durability of concrete structures to reduce the production of raw materials and natural resources [7]. From the perspectives of infrastructure management agencies, condition assessment of concrete in structures is important to monitor the severity of deterioration, and if necessary, to make an appropriate maintenance action [8], which prolongs the service life of concrete, thereby reducing the environmental impact on the society.

Electrical resistivity (ER) method is among the most popular non-destructive evaluation (NDE) techniques for in-situ evaluation of durability performance of concrete because of its simplicity, speed, and low-cost during field inspections [9,10,11,12]. Some organizations uses the ER measurements for construction and maintenance procedures [13,14]. It is discussed in prior studies that ER can be correlated to corrosion rate [15,16,17,18,19] and chloride diffusivity [20,21,22,23,24]. By definition, electrical resistivity is generally a means of measuring the water content and micropore structure of a concrete structure, hence making it good in analyzing concrete’s strength and durability [12]. It is also defined as the ability and potential of the cementitious material to withstand and resist the transfer of ions from an external electric current applied to its surface [25,26].

However, special cares are needed to obtain reliable and accurate interpretation of ER data in concrete. It is mainly because ER values of concrete are sensitive to various parameters related to material properties of concrete and environmental factors, which include water/cement ratio [27,28], age of concrete [29,30], moisture content and degree of saturation [31], specimen geometry [32], temperature [33,34], electrode spacing [35], and presence of re-bars [25,36]. The concrete’s microstructure properties such as the volume and pore size have a direct effect on resistivity measurements [37]. For instance, a number of studies have observed that the degree of saturation can affect the ER measurements. The increase of water content in concrete significantly decreases ER values [38,39,40,41,42]. Researchers also pointed out that the variation of the ER values could be caused by different factors such as the rebar diameter and spacing, the electrode location and configuration, orientation of the rebar with respect to the probe, and the thickness of the concrete cover. [43,44,45].

**Figure 1:**
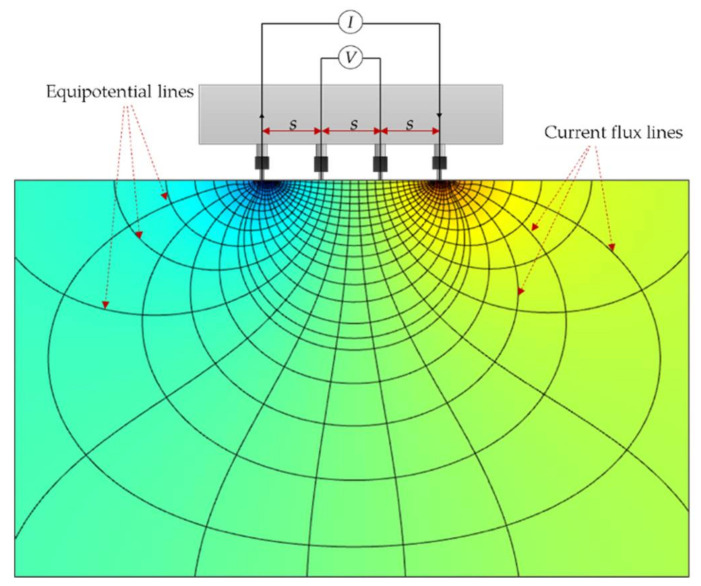
Wenner probe configuration, with ‘s’ as the distance between the electrodes.

Specifically, a functional relationship between the apparent resistivity of concrete and its shape/geometrical composition should always be considered [46,47,48]. In principle, electrical resistivity (ρ) is the quotient of the potential difference over the applied electric current, multiplied by a geometric constant. In a Wenner probe configuration, four electrodes are aligned in a linear manner at an equal distance, s, to each other as illustrated in Figure 1. The external current (AC current), I, is imposed at the two exterior electrodes and the electrical potential difference, V, is measured through two internal electrodes [49]. Using the Wenner probe method, the measurement of the concrete resistivity is assumed to be on a semi-infinite, isotropic, and homogenous medium [50,51]. Mathematically, the apparent resistivity ($\rho_{app}$) of concrete can be expressed as Equation (1). Considering this equation, the ER measurement does not include the parameters of thickness and the surface area of the concrete specimen [46]. For example, for relatively small specimens such as beams and cylinders, constriction in the flow of the current results in an overestimation of the ER measurements. Spragg et al. (2013) [41] suggested a correction coefficient to account for the interference caused by the geometrical constraints of small concrete samples which was based on the numerical simulation conducted by Morris et al. (1996) [52]. It is defined that the true resistivity (ρ) is equal to the ratio of the apparent resistivity (ER value shown on display screens of commercial Wenner probe devices) and the geometrical correction factor, *K* (see Equation (2)).

(1) $$\rho_{app}=2\pi s\;\left(\frac{V}{I}\right)$$

(2) $$\rho =\frac{\rho_{app}}{K}$$

This correction factor varies and is dependent on the sample’s thickness, geometric dimensions, surface area, and probe configuration [53]. However, most research in the literature focused on cylindrical concrete samples. Only limited published papers discussed other geometric shapes. Specifically, few studies investigated the effect of concrete slabs on ER measurements. Garzon et al. (2014) devised a mathematical expression through numerical simulation for the shape factor of a large slab with unknown dimension and only considering an electrode spacing of 35 mm. It is emphasized in the study that the shape factor is dependent on the rebar spacing [40]. Gowers and Millard (1999), through their experimental investigation, summarized their recommendations on the proper application of the Wenner probe method on slabs. The probe spacing should be less than or equal to ¼ of the cross section’s thickness and lateral dimension (perpendicular to probes), greater than 40 mm, and less than or equal to 2/3 of the concrete cover [43]. Bryant et al. (2009), in their study, pointed out that for the same concrete design mix, cylindrical specimens showed a higher resistivity than concrete slabs [29]. In addition, Chen et al. (2014), in their experimental investigation, suggested that the correction factor be based on the ratio of the length of concrete specimen to electrode spacing. No study was performed for both larger concrete specimens, and for samples with more than one steel reinforcement.

The main objectives of this research are to evaluate the effect of geometrical constraints of plain concrete and reinforced concrete slab on the electrical resistivity measurements through numerical and experimental investigation and to propose measurement recommendations for laboratory and field specimens. This research will discuss the effect of the geometrical constraints in the ER measurements such as the dimensions of concrete slabs, the electrode spacing and configuration, and the distance of the electrode to the edges of concrete slabs. This study will also analyze the effect of the presence of steel reinforcement and concrete saturation condition. The findings in this study will further improve the understanding of the geometrical constraint effect on ER values measured on the regularly shaped concrete elements and enable more reliable and accurate interpretation of ER values in the laboratory and field applications.

## 2. Methods

### 2.1. Numerical Simulation

**Figure 2:**
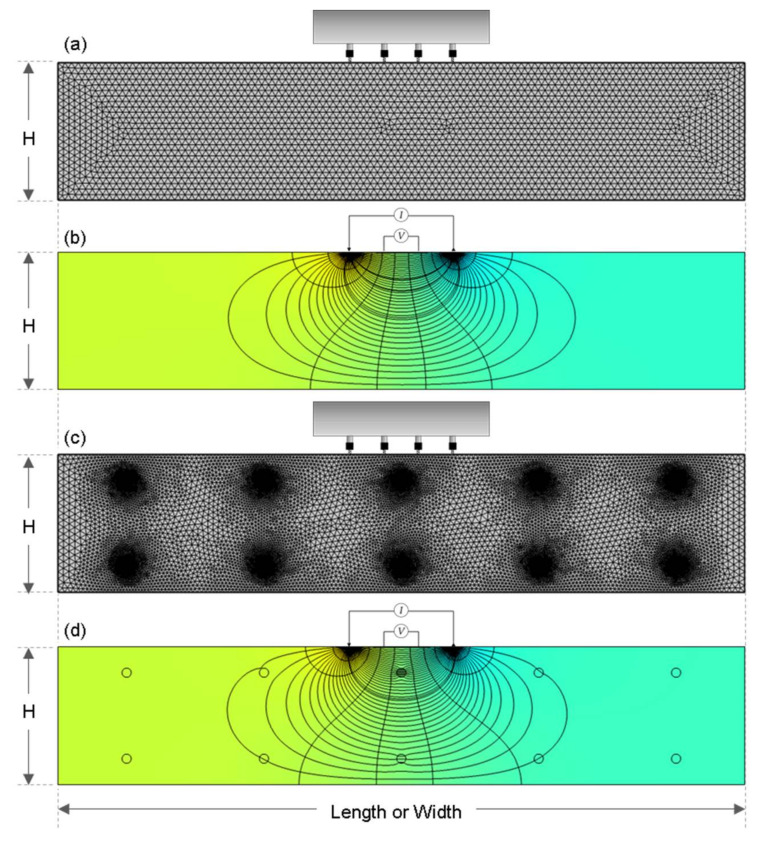
2D cross-section of finite element models for simulation of electrical field in concrete: (**a**,**c**) represents a finite element model for a plain and a reinforced concrete slab, respectively; (**b**,**d**) for the electric potential field in a plain and a reinforced concrete slab, respectively.

A 3D finite element model was developed to investigate the variation of electric potential field in concrete slabs with various geometrical dimensions using a commercially available code, AC/DC module in the COMSOL Multiphysics v5.5. Figure 2 illustrates the mesh analysis and the results of the electric potential distribution of the simulated concrete slab. The electric potential computation for this simulation followed the principle of the classical Poisson’s equation derived using Gauss law and equation of continuity as follows,

(3) $$-\nabla \cdot \left(\sigma \nabla V-J^{e}\right)=Q_{j}$$

where σ is the electrical conductivity (inverse of ER), $J^{e}$ is the externally generated electric current, and $Q_{j}$ is the current source. This simulation consists of tetrahedral and triangular mesh elements ranging from 0.004 cm–0.4 cm per mesh element with an average mesh density of 60,394,510 mesh elements/m^3^.

Using an input true resistivity of 100 kΩ-cm and an external current of 200 μA placed at the two external electrodes, the electric potential difference (in V) was measured using two boundary probes placed at the location of the two internal electrodes. Similar to the four-point Wenner probe, the four electrodes had an equal spacing (s) in the numerical simulation models. For comparison to the experimental data, the simulated potential difference was used to compute for the apparent ER using Equation (1).

**Figure 3:**
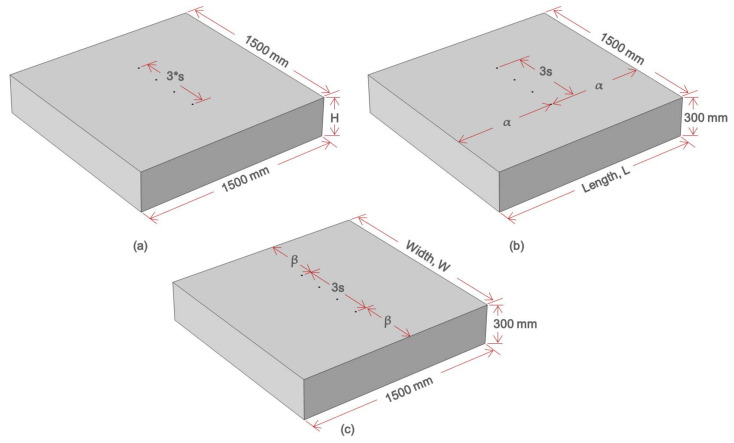
Concrete model with different geometric and probe configuration for the three simulation cases: (**a**) Case 1, (**b**) Case 2, and (**c**) Case 3.

In this study, a series of numerical simulations was conducted to investigate the effect of geometrical properties and constraints of both the plain and reinforced concrete slabs to the measurement of electrical resistivity of concrete. Main variables in the numerical simulation included the dimensions of concrete slabs (slab height, width, and length), the spacing of sensors (electrodes), and the presence of reinforcing steel in concrete slabs. The variables are summarized in Table 1 and illustrated in Figure 3.

Figure 3a–c show the three different numerical simulation models of plain concrete slabs with various slab thicknesses and locations of electrode probes relative to the edge of concrete slabs. Figure 3a represents the simulation case 1 where the variable in consideration is the slab height, H. The simulation cases 2 and 3 are shown in Figure 3b,c illustrating the simulation of varying length and width, respectively.

For all the numerical simulation models, three different electrode spacings were used to investigate the effect of the spacing of electrodes on the apparent ER measurements. To analyze the effect of increasing electrode spacings and to simulate the commercially available Wenner Probe devices, probe spacings, s of 38 mm, 50 mm, and 76 mm are used. In addition, another set of numerical simulation was performed by using reinforced concrete slabs to evaluate the effect of reinforcing steel on the ER measurements as shown in Figure 2c. The two layers of rebar mesh were placed 50 mm from both the surfaces of the slab.

### 2.2. Experimental Study

#### 2.2.1. Preparation of Concrete Slab Specimen

**Figure 4:**
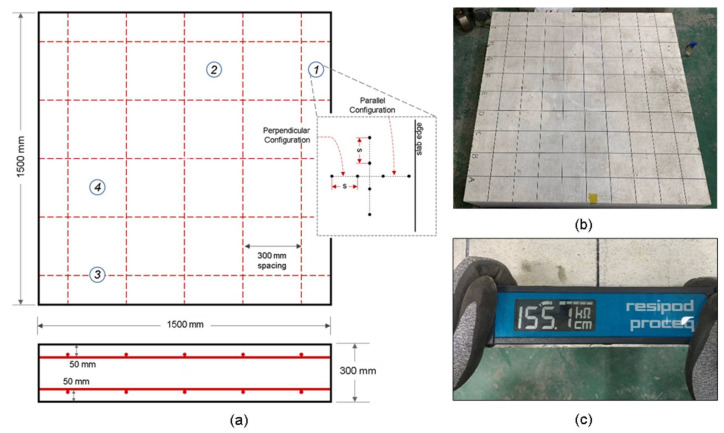
Experimental set-up of ER measurement in concrete slab: (**a**) schematic diagram of concrete slabs with measurement points; (**b**) picture of actual specimen; and (**c**) Wenner probe device orientation during ER measurements.

A reinforced concrete slab with 1500 mm length, 1500 mm width, and 300 mm height manufactured at Dong-A University was used for this experiment. The concrete used for the fabrication of the concrete slabs was composed of Type I Portland cement, river sand and crushed coarse aggregate, and water. The mixture was designed to 28-day compressive strength of 35 MPa. Table 2 summarizes the material properties and composition of the concrete specimen. As shown in Figure 4, two layers of 13-mm diameter uncoated steel mesh were placed with a 300 mm center-to-center spacing. The top and bottom layers were placed 50 mm and 250 mm from the concrete top surface, respectively. In order to keep it in air-dried condition, the concrete specimen was kept in the laboratory with room temperature of 20 ± 3 °C.

#### 2.2.2. Electrical Resistivity Measurements

A commercially available Wenner Probe device (Resipod Proceq), with an electrode spacing of 38 mm, was used in measuring ER at the surface of the reinforced concrete slab as shown in Figure 4c. The device follows the standard specification for AASHTO Designation T358-15 (Surface Resistivity Indication of Concrete’s Ability to Resist Chloride Ion Penetration) [54]. Depending on the concrete’s contact resistance, an input current with a minimum value of 10 μA up to a maximum value of 200 μA is driven to the concrete from its surface [55]. The device displays an output value in in kΩ-cm, the unit of measurement for apparent electrical resistivity.

The measurement locations for gathering the ER values of the reinforced concrete slab are shown in Figure 4a. For evaluating the edge effect in ER measurements, point ① and point ② were established to compute for the relative electrical resistivity (ratio of apparent ER at edge of slab over apparent ER of solid concrete slab). Point ① is located near the edge and at equal distance from 2 parallel rebars; and point ② is located farthest from the rebar mesh. For evaluating the effect of the edge of the slab, the Wenner device was initially placed 10 mm away from the edge (see Figure 4c) with an increment of 10 mm and farthest measurement location of 150 mm away from the edge where the probe configuration was parallel to the edge of the slab. Five measurements were performed at each location. The experiment was repeated where the probes were perpendicular to the slab edge.

It is of importance to figure out the effect of saturation conditions on ER measurements on concrete slabs since ER of concrete is strongly dependent on water content in concrete. To evaluate and minimize the effect of the degree of saturation of concrete, the electrical resistivity of concrete was measured in three saturation conditions: no saturation, one-day saturation, and two-day saturation of the surface of the concrete. ER values of dried concrete are extremely high and often exceed the capacity of measurement devices. Accordingly, a number of researchers specified to wet the surface by using a sponge or spraying a small amount of water before making a measurement [37,56,57,58,59]. However, there has been no standard guide for determining the amount of water (or standard saturation condition) for ER measurement. In this study, the relative ER (ratio of the apparent ER measured directly above the rebar over the apparent ER of solid concrete slab) of concrete was measured at the point ③ and point ④ as measurement locations to investigate the effect of saturation conditions and the presence of rebars to ER measurements. The probes were placed parallel to the horizontal axis and the resistivity were measured every 4 min. Instantaneous saturation of the concrete surface was measured for a total duration of 50 min. The locations of the rebars were determined using a portable GRP system (StructureScan Mini XT produced by Geophysical Survey Systems Inc. (GSSI)).

## 3. Results and Discussion

### 3.1. Variation of Geometrical Correction Factor of ER in the Plain Concrete Slab Models

**Figure 5:**
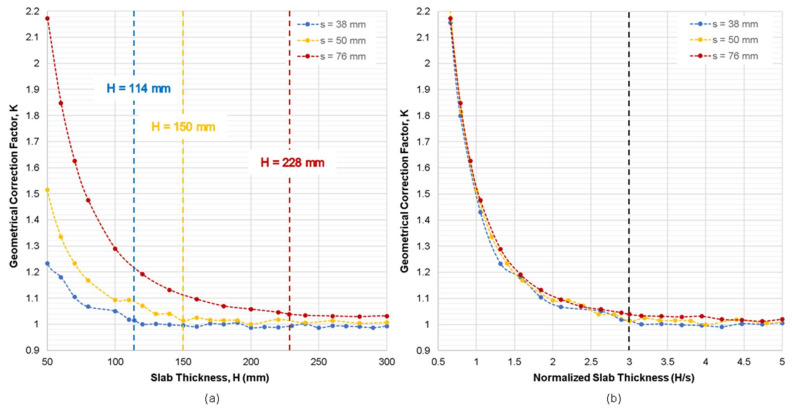
Variation of geometrical correction factor, *K*, obtained from the numerical simulation case 1 for the plain concrete slab model with respect to: (**a**) slab thickness, *H*, and (**b**) normalized slab thickness (*H*/s).

Figure 5a shows the variation of geometrical correction factor, *K*, of the plain concrete model with varying slab thickness, *H* (see Figure 3a), measured by the three different electrode spacings (s = 38 mm, 50 mm, 76 mm). The geometrical correction factor, *K*, was defined as the apparent ER values obtained by Equation (1) over the true ER values for the simulation model (100 kΩ-cm in this study). It is observed that the *K* value is dependent of slab thickness and electrode spacing. For the same electrode spacing, the value of *K* decreases as the slab thickness increases. For the same slab thickness, greater electrode spacing results in greater *K* value. In addition, Figure 5b shows the variation of *K* value with normalized slab thickness (*H*/s), the slab thickness *H* divided by the electrode spacing, s. Three graphs from different electrode spacings shows a good agreement with each other, which demonstrates that the geometrical correction factor, *K*, is mainly dominated by the normalized slab thickness, *H*/s, for the plain concrete model. This supports the results of previous researches that as the probe spacing increases, a thicker region of concrete would be involved in ER measurements [50,59]. Based on the simulated results, it can be concluded that the normalized thickness should be at least 2.0 (*H* ≥ 2 s) to minimize the correction factor less than 10% (*K* ≤ 1.1). Furthermore, the normalized thickness should be greater than 3.0 to completely suppress the thickness effect on the ER measurements (*H* ≥ 3 s). This results of this part of the also validates the findings of Gowers and Millard (1999) and Yilmaz (2015) that as the thickness increases, the geometrical correction factor decreases [43,46].

**Figure 6:**
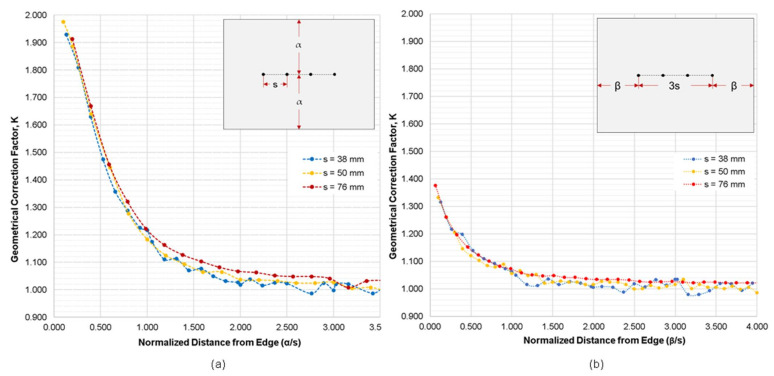
Variation of geometrical correction factor, *K*, obtained from the numerical simulation cases 2 and 3, with respect to the normalized distance from slab edge where (**a**) probes are parallel to the edge of slab, and (**b**) probes are perpendicular to the slab edge.

Figure 6a,b show the variation of geometrical correction factor, *K*, obtained from the numerical simulation cases 2 and 3 for the plain concrete model with increasing the normalized distance of a probe array from a slab edge, α/s and β/s, respectively. This follows the recommendation of Chen et al. (2014) that the geometrical correction factors should be based on the ratio of the length of the specimen over the electrode spacing [12]. Note α and β represent the respective parallel and perpendicular distances of the probes from the slab edge (see also the probe configuration in inserted figures in Figure 6a,b). The results of these simulations demonstrated that as the gap between the probes and the concrete edge increases, the geometric correction factor, *K*, decreases and becomes steady. To reduce the correction factor to less than 10% (*K* ≤ 1.10), α/s and β/s should have a minimum value of 1.58 and 0.72, respectively. In particular, an ER probe should be located farther away from a slab edge, with the α/s and β/s greater than 2.0, to suppress the *K* values less than 5% (*K* ≤ 1.05). It should be taken into account that other material properties and environmental factors such as the presence of rebars and degree of saturation in concrete were not considered in this part of the analytical study.

### 3.2. Variation of Geometrical Correction Factor of ER in the Reinforced Concrete Slab Models

**Figure 7:**
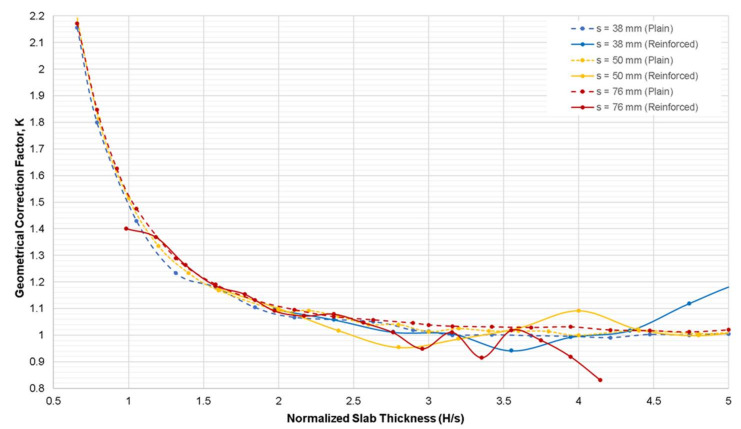
Variation of geometrical correction factor, *K*, obtained from the numerical simulation case 1 for the reinforced concrete slab model with respect to the normalized slab thickness (*H*/s).

Figure 7 shows the variation of geometrical correction factor, *K*, obtained from the numerical simulation case 1 using reinforced concrete slab models with respect to the normalized slab thickness (*H*/s). The orientation of the two layers of steel reinforcement was arranged in the reinforced concrete slab models in accordance with the actual concrete slab shown in Figure 2. The concrete cover of the rebars was fixed at 50 mm at both surfaces of the slab. For comparison, the simulation results obtained from the plain concrete are shown with dashed lines in the same figure. Figure 7 depicts that the presence of rebar in concrete is a critical factor in the measurement of the electrical resistivity of concrete. Plain concrete simulation shows a downward trend of *K* value with increasing thickness. However, simulation of reinforced concrete shows that both the slab thickness and steel reinforcements affect the *K* values. It can be interpreted that for the normalized slab thickness of reinforced concrete less than 2.0 (*H*/s < 2.0), the governing factor on the relative ER is the slab thickness, where it shows the similar trend as the relative ER of plain concrete. In contrast, for *H*/s ≥ 2.0, fluctuating values in ER were observed, attributed to the disturbance of current flow due to the presence of rebars in concrete. Different electrode spacings show different trends. There have been intensive studies on the effect of the presence of rebars in concrete on the electrical resistivity measurements of concrete through experimental and numerical investigation [25,36,43,44,45,60]. It is explained that the embedded rebar in concrete causes the distortions on the electrical current fluxes produced by the probes [25,36]. Researches pointed out that the alteration of ER values are caused by various factors such as the rebar diameter and spacing, the electrode location and configuration, orientation of the rebar with respect to the probe, and the thickness of the concrete cover [43,44,45]. This study used only one rebar mesh setup and focused on the effect of the slab thickness. Based on the results in this study, it appeared that the electrode spacing, the slab thickness, and steel reinforcement all contributed to the variation of the relative ER values of concrete. Considering both the effect of rebar and slab thickness requires a more detailed study.

**Figure 8:**
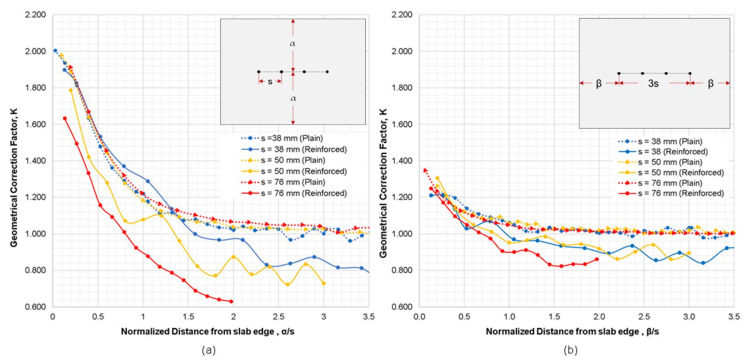
Variation of geometrical correction factor, *K*, obtained from the numerical simulation cases 2 and 3 for the reinforced concrete models with respect to the normalized distance from slab edge where (**a**) probes are parallel to edge of slab, and (**b**) probes are perpendicular to the slab edge.

Figure 8 represents the variation of geometrical correction factor, *K*, obtained from the numerical simulation cases 2 and 3 for the reinforced concrete models with respect to the normalized distances from slab edge, α/s and β/s, respectively. Figure 8a,b show a clear gap between the simulation results conducted for the plain concrete slab models (dotted lines) and the reinforced concrete models (solid lines). It is observed that the *K* values obtained on the reinforced concrete models were lower than those obtained from the plain concrete models. The differences in the *K* values of different electrode spacings range from 22% to 40% (a parallel configuration in Figure 8a) and 11% to 15% (a perpendicular configuration in Figure 8b) lower than the relative ER simulated of plain concrete. This shows that the perpendicular configuration of electrodes with respect to slab edge results in a smaller difference of *K* values between plain and reinforced concrete models, as compared to the results of the parallel configuration. It can also be inferred that for the simulation of both the plain and reinforced concrete models, the change of β (concrete slab width perpendicular to the electrode array) has less effect on size correction factor *K* compared to the change of α (concrete slab width parallel to the electrode array).

Interestingly, it can also be deduced that a normalized graph was not formed in the numerical simulation for the reinforced concrete models. The result of the numerical simulation for reinforced concrete model showed that the increasing electrode spacing resulted to lower *K* values. It was observed that the relative ER generated through numerical simulation for a 76 mm electrode spacing yielded lower resistivity values as compared to 38 mm and 50 mm electrode spacings. The simulation using an electrode spacing of 38 mm produced a graph closest to the normalized graph of plain concrete, whereas 76 mm probe spacings produced the farthest. It can be interpreted that larger probe spacings cover more rebars, resulting in lower relative ER values. Therefore, the location of the rebar and its orientation with respect to the probe has a significant effect to ER measurements, as mentioned in previous paragraphs.

### 3.3. Comparison of Numerical Simulation and Experimental Studies

**Figure 9:**
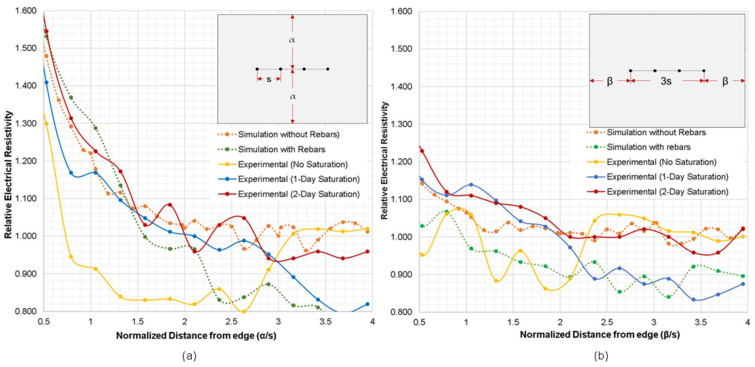
Numerical and experimental relative electrical resistivity of concrete slab with respect to the normalized distance from edge of slab, where (**a**) probes are parallel to edge of slab, and (**b**) probes are perpendicular to slab edge.

**Figure 10:**
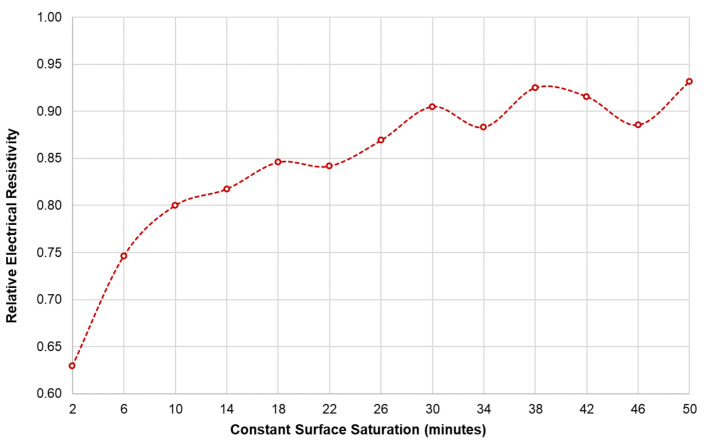
Variation of the relative ER values measured directly above a reinforcing bar in concrete specimens with respect to the surface saturation of concrete.

**Figure 11:**
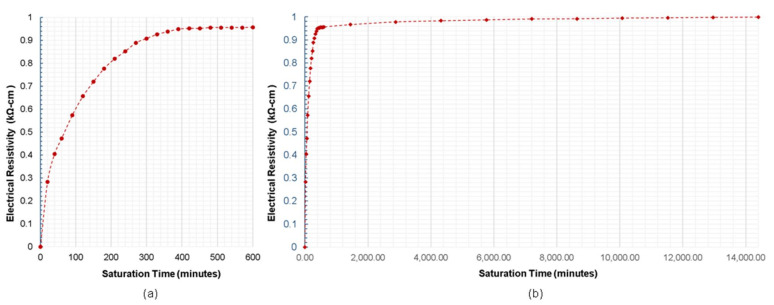
Relationship of the surface saturation of concrete with respect to the relative ER measured directly above the rebar with saturation time of (**a**) up to 600 min (**b**) 14,400 min (10 days).

To fully understand the edge effect of plain and reinforced concrete slabs, an experimental investigation was also conducted considering an electrode spacing of 38 mm. In this part of the study, instead of using the geometrical correction factor, *K*, as variables for comparing the experimental and numerical data, the relative ER (ratio of apparent ER measured at the slab edge over apparent ER of solid concrete) is used. With reference to Equation (2), the geometrical correction factor of the reinforced concrete specimen cannot be derived since the Wenner device only displays the apparent ER and not the true ER of concrete. Figure 9 shows the relationship between the relative ER and the distance of the probes from the edge of the slab gathered using both experimental investigation and numerical simulation. Using two sets of probe configurations for this experiment (see Figure 4a), three sets of measurements were performed: no surface saturation; one-day partial surface saturation; and two-day partial surface saturation. It can be inferred in both graphs that for the measurements performed with the air-dry condition, no relationship and similarity could be seen between the numerical and experimental data. For a specimen with very little moisture concrete, ER measurement would be unreliable [61]. On the other hand, both the relative ER of the one-day and two-day partial surface saturation measurements show a downward trend. The trend of the relative ER for the two-day saturation is comparable to the trend of the relative ER obtained from the numerical simulation for the plain concrete model. It can be interpreted in this results that increasing the degree of saturation of the reinforced concrete decreases the effect of the steel reinforcement to the measurement of electrical resistivity. This result is supported by Figure 10, showing the effect of the surface saturation of concrete to the relative ER measured directly above the rebar. At the first 2 min, there is an approximately 37% (relative ER of 0.63) difference between measurements done above the rebar and the solid concrete. The relative ER increases to 0.93 after 50 min of instantaneous saturation. It can be concluded that as the water content of concrete increases, the effect of steel reinforcement to ER measurement decreases. The data presented with in the experimental study validates the conclusion of prior researches that the degree of saturation is a vital parameter to be considered in ER measurement, whereas as the degree of saturation increases, the ER decreases and becomes constant when it is fully saturated (as shown in Figure 11) [37,38,62]. However, in Figure 10, a fluctuation of relative ER values is observed after 30 min of instantaneous saturation. It is safe to conclude that the degree of saturation, concrete composition and the presence of rebar contribute to this result. It should be noted that this part of the study is limited to the surface saturation of concrete. Considering the result of both Figure 10 and Figure 11, it is reasonable enough to infer that the degree of saturation has an increasing relationship to relative ER of concrete influenced by the presence of rebar. This is consistent with the results of the experimental study conducted by Morales (2014) that the relative ER measured at the top of the rebar increases as the degree of saturation increases [63]. More so, consistent with the numerical simulation, the experimental data shows that in order to disregard or minimize the edge effect, the distance of the probe from the edge should be more than twice the electrode spacing (e = 2 × s). Figure 9 shows that for both numerical simulation and experimental investigation (2-day saturation), the governing factor for ER measurements for α/s and β/s lower than 2.0 is attributed to the edge effect.

### 3.4. Guideline for ER Measurement in Regularly Shaped Reinforced Concrete Elements

**Figure 12:**
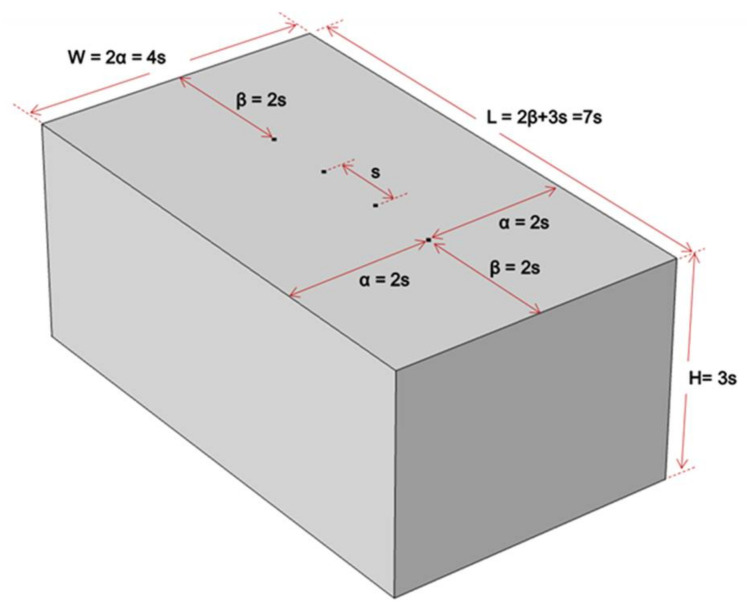
Schematic illustration for ER measurement recommendations.

The findings of the experimental investigation and numerical simulation of reinforced concrete slab confirmed that the ER probe should be located far enough away from the bottom and the edges of concrete elements to reduce the geometrical constraint effect on ER measurements, consistent with the recommendations of existing studies [12,43]. The analytical and experimental study provide evidence that the numerical simulation of plain concrete can be a good reference for geometrical correction factor recommendations. As a rule of thumb, it is recommended that the ER measurements be measured on a concrete slab with a thickness greater than at least three times electrode spacing (H ≥ 3 × s), and the ER probe be located far away at least two times electrode spacing from the slab edges (α/s and β/s ≥ 2.0). A schematic diagram, as inspired by the paper of Gowers and Millard (1999) [43], is illustrated in Figure 12, indicating the recommendations for the minimum dimensions of a concrete slab to have the minimum geometrical correction factor (*K* ≈ 1.0). For a commercially available Wenner probe device with an electrode spacing, s = 38 mm, the minimum value for both α and β is 76 mm. This also means that the minimum dimensions (L × W × H) for a concrete slab is 266 mm × 152 mm × 114 mm. This slab dimensions when simulated in COMSOL generates a geometrical correction factor of 1.0032, validating the recommendation of this study.

It was observed in this study that the apparent ER values are strongly affected by the presence of reinforcing bars when ER measurements are done on the reinforced concrete slabs. The results from numerical simulations and experiments show that the guideline to avoid the geometrical constraint effects for the plain concrete may not be effective for the reinforce concrete slabs.

One interesting finding in this study is that concrete in the wet condition decreased the disruptions caused by the presence of steel reinforcement. As discussed, Figure 9a,b show that for one-day and two-day partial saturation of concrete, ER measurements done at a distance of less than twice the electrode spacing from the edge of the slab (α and β < 2 s), the geometrical configuration of slab governs more than the degree of saturation and rebar presence in the variation of relative ER, whereas for α and β > 2 s, relative ER measurements was affected mostly by the rebar presence and the concrete’s saturation condition. Figure 11 shows the trend of the degree of saturation of a concrete specimen with respect to instantaneous surface saturation. With this and Figure 10 as references, the variance percentage of the relative ER can be estimated at different saturation degrees. If this is to be applied in field inspection for instance, the inspectors/engineers have to saturate the surface of concrete slabs (e.g., pavements and bridge decks) for approximately 20 min, to minimize the relative ER to 15% (with a degree of saturation of 0.28). Moreover, for 50 min immersion of concrete, a seven percent decrease is observed. Still, considering both the degree of saturation and presence of steel reinforcement in ER measurement is complicated and needs further study.

## 4. Conclusions

The effects of geometrical constraints of plain concrete and reinforced concrete slab to the electrical resistivity (ER) measurements are evaluated in this research study. The researchers utilized the four-point (Wenner Probe) method in determining the electrical resistivity of a reinforced concrete specimen. A numerical simulation through COMSOL Multiphysics was also conducted to be compared to the experimental data. Generally, it can be concluded, that the slab dimensions (length, width, and thickness) contribute to the varying geometrical correction factor of the specimen. The specific conclusions and recommendations derived from this study are summarized below:

1. Numerical simulation shows that to minimize the effect of the slab thickness in the measurement of apparent electrical resistivity, the thickness of the concrete slab should be three times the electrode spacing (*H* ≥ 3 × s). Using numerical simulation of plain concrete model, to make the edge effect almost negligible, the distance should be more than twice the electrode spacing (α and β ≥ 2 × s).
2. For the simulation of reinforced concrete slab, the presence of rebars causes disruption to the current flux lines that results to the fluctuations on the resistivity values. Based on the results gathered, it appeared that the electrode spacing, the slab thickness, and steel reinforcement all contributed to the variation of the relative ER values. Numerical simulation of reinforced concrete model shows significantly lower relative ER values as compared to that of the plain concrete model. The larger the electrode spacing, the lower the relative ER for reinforced concrete.
3. The experimental investigation of the edge effect shows that a two-day surface saturation of the reinforced concrete specimen results to a trend of the relative ER consistent with the plain concrete model simulation. No trend has been observed for the slab in the air-dried condition. It is concluded in this experiment that increasing the degree of saturation of concrete decreases the effect of rebar in ER measurements. Experimental investigation also concluded that for α and β ≤ 2 × s, the governing factor for the relative ER is the geometrical composition of the slab. Otherwise, presence of rebar and degree of saturations should be primarily considered in ER measurements.
4. For field investigations, to minimize the effect of the presence of steel reinforcements, it is recommended to saturate the surface of the concrete slab for around 20 min to have a minimum relative ER of approximately 0.85. For accurate data analysis, extreme caution and care is needed during ER measurements.
5. A schematic diagram of the recommended minimum dimension of a concrete to have the least geometric correct factor is shown in Figure 12.

## Figures and Tables

**Table 1:** Main variables of the numerical simulations in this study.

Simulation Case	Dimensions of Concrete Slabs (Intervals) [mm]			Location of an Electrode Array (Intervals) [mm]		Electrode Spacing [mm]
	Slab Height, H	Length, L	Width, W	α	β	s
Case 1	50~300 (10)	1500	1500	750	750–1.5 × s	38, 50, and 76
Case 2	300	2 × α	1500	10–450 (10)	750–1.5 × s	
Case 3	300	1500	3 × s + 2 × β	750	10–450 (10)	

Note: α = distance of the center of the electrode to the edge of the concrete slab perpendicular to the electrode array, β = distance of the external electrode to the edge of the concrete slab parallel to the electrode array, s = electrode spacing. Values of L, W, α, and β are dependent to the electrode spacing, s.

**Table 2:** Properties of the concrete cylinders used in this study.

W/C	S_V_/A_V_	Mixture Proportion [kg/m^3^]				
		W	C	S	G	AE
0.375	0.405	165	440	701	1049	3.08

Note W/C: water-to-cement ratio, S_V_: volume of sand, A_V_: volume of aggregates, W: water, C: Portland cement type I, S: sand, G: gravel, AE: high performance air-entraining agent.

## Data Availability

Data are contained within the article. But the data presented in this study are also available on request from the corresponding author.
